# The Association between Intrauterine Inflammation and Spontaneous Vaginal Delivery at Term: A Cross-Sectional Study

**DOI:** 10.1371/journal.pone.0006572

**Published:** 2009-08-10

**Authors:** Michiel L. Houben, Peter G. J. Nikkels, Grada M. van Bleek, Gerard H. A. Visser, Maroeska M. Rovers, Hilda Kessel, Wouter J. de Waal, Leontine Schuijff, Annemiek Evers, Jan L. L. Kimpen, Louis Bont

**Affiliations:** 1 Department of Pediatrics, Wilhelmina Children's Hospital, Utrecht University Medical Center, Utrecht, The Netherlands; 2 Department of Pathology, Utrecht University Medical Center, Utrecht, The Netherlands; 3 Department of Gynecology and Obstetrics, Wilhelmina Children's Hospital, Utrecht University Medical Center, Utrecht, The Netherlands; 4 Department of Epidemiology, Utrecht University Medical Center, Utrecht, The Netherlands; 5 Department of Gynecology and Obstetrics, Diakonessen Hospital, Utrecht, The Netherlands; 6 Department of Pediatrics, Diakonessen Hospital, Utrecht, The Netherlands; National Institute of Child Health and Human Development, United States of America

## Abstract

**Background:**

Different factors contribute to the onset of labor at term. In animal models onset of labor is characterized by an inflammatory response. The role of intrauterine inflammation, although implicated in preterm birth, is not yet established in human term labor. We hypothesized that intrauterine inflammation at term is associated with spontaneous onset of labor.

**Methods/Results:**

In two large urban hospitals in the Netherlands, a cross-sectional study of spontaneous onset term vaginal deliveries and elective caesarean sections (CS), without signs of labor, was carried out. Placentas and amniotic fluid samples were collected during labor and/or at delivery. Histological signs of placenta inflammation were determined. Amniotic fluid proinflammatory cytokine concentrations were measured using ELISA. A total of 375 women were included. In term vaginal deliveries, more signs of intrauterine inflammation were found than in elective CS: the prevalence of chorioamnionitis was higher (18 vs 4%, p = 0.02) and amniotic fluid concentration of IL-6 was higher (3.1 vs 0.37 ng/mL, p<0.001). Similar results were obtained for IL-8 (10.93 vs 0.96 ng/mL, p<0.001) and percentage of detectable TNF-α (50 vs 4%, p<0.001).

**Conclusions:**

This large cross-sectional study shows that spontaneous term delivery is characterized by histopathological signs of placenta inflammation and increased amniotic fluid proinflammatory cytokines.

## Introduction

Normal human delivery is initiated by the spontaneous onset of labor at term.[1] The role of inflammation in the onset of labor at term in humans is poorly understood, although it has been implicated in the physiological onset of parturition.[2]–[5] Inflammation at term comprises the gold standard of histologically proven infiltration of the chorioamniotic tissue, but also presence of inflammatory mediators in the amniotic fluid.[6] Microbial invasion of the amniotic space is found in up to 19% of term pregnancies.[7] However, infection contributes only to a small proportion of cases with signs of chorioamnionitis; amniotic fluid appears sterile in the majority of these cases. In general, histopathological inflammation, including chorioamnionitis develops sub-clinically.

The clinical relevance of sterile inflammation in normal delivery is intriguing. Inflammation may provide a danger signal by the innate immune system, resulting in the onset of labor.[8] Pro-inflammatory molecules stimulate the maternal myometrial cells and induce cerival ripening.[1] There is evidence in literature showing that sterile inflammation at term is indeed associated with the onset of labor in the animal model.[9]–[12] The relevance of these findings for humans is not clear, since mechanisms of parturition are species-dependent. Human data showing that inflammation is associated with term delivery are rare.[6], [7], [13], [14] In the current study we aimed to establish the relationship between peripartum signs of inflammation and spontaneous onset of term delivery in humans. We hypothesized that intrauterine inflammation at term is associated with spontaneous onset of labor. We compared the inflammatory response in normal vaginal deliveries with elective caesarean sections (CS) at term. To our knowledge, this is the first large cross-sectional study of normal term deliveries integrating both histological studies and amniotic fluid cytokine measurements, showing extensive inflammation in placenta tissue and in amniotic fluid in women with spontaneous onset of labor.

## Methods

### Study population

A cross-sectional study was conducted in one secondary and one tertiary hospital in Utrecht, The Netherlands, between January 2006 and November 2007. Women delivering vaginally at term after an uncomplicated pregnancy and with spontaneous onset of labor were recruited. In addition, women who delivered by elective CS were recruited. Elective CS was defined as a planned CS, performed in the absence of signs of labor or rupture of membranes. Women were excluded if they did not comprehend the Dutch language, if the gestational age was below 37 weeks, or if the infant had major congenital organ abnormalities (such as spina bifida and congenital heart disease). Recruitment was performed by one of the researchers. Shortly before or after delivery, parents were informed about the study. Collected samples of the placenta and amniotic fluid were only analysed when parents consented. The study was approved by the ethical review boards of both institutions and all parents provided written informed consent for study participation.

### Clinical characteristics

Clinical characteristics of the mother (maternal age, gestational age, parity, meconium stained amniotic fluid, fever during delivery, assisted delivery, antenatal corticosteroid administration) and her child (birth weight, gender, Apgar score five minutes after birth, newborn infection) were collected. Gestational age was determined with the use of clinical history and the results of the earliest ultrasound examination. Secondary CS was defined as a CS performed after the onset of labor. Maternal fever during delivery was defined as a temperature of 38.0°C or higher, as measured during labor and delivery. Newborn infection was defined as a strongly suspected or proven early onset neonatal sepsis, within 48 hours after birth.

### Collection of samples

Placentas were stored at +4°C and processed within 72 hours. In case of vaginal delivery, amniotic fluid was collected vaginally, in a non-sterile manner, at the moment of artificial rupture of membranes or after spontaneous rupture of membranes. When spontaneous rupture of membranes occurred outside the hospital, collection of amniotic fluid was usually performed immediately after birth, because at that moment a larger volume of amniotic fluid passes the vagina. During elective CS, amniotic fluid was collected with a syringe directly after incision of the membranes. Amniotic fluid samples were stored at +4°C and processed within 72 hours.

### Placenta histology

Placenta examination was performed by an experienced pathologist (PN).[15] The pathologist was masked for clinical information. Two sections of umbilical cord, at the fetal and placental side, a membrane roll, one sample from the umbilical cord insertion, and three slides of normal placental parenchyma, including both decidua and chorionic plate, were collected and stained with standard haematoxylin and eosin. Histological chorioamnionitis was diagnosed based on the presence of polymorphonuclear cells (neutrophilic granulocytes) in the chorionic plate or the extraplacental membranes. Funisitis was diagnosed in the presence of neutrophils in the wall of the umbilical vein and/or arteries or Wharton's jelly and villitis was diagnosed as an infiltration of lymphocytes and macrophages in the placental villi. In addition to the presence or absence of chorioamnionitis, funisitis or villitis, the severity of inflammation was graded mild, moderate or severe, with slight modifications comparable to the staging and grading system of Redline (table 1).[16]

**Table 1:** Histological classification of chorioamnionitis, funisitis and villitis.

Grade	Chorioamnionitis	Funisitis	Villitis
0	No inflammation	No inflammation	No inflammation
0.5	Sporadic PMN in chorionic plate/membranes		
1	Frequent PMN in chorionic plate/membranes	Inflammation present in the wall of 1 vessel (vein)	1 section with 1 focus of chronic inflammation of >5 villi
2	Invasion of chorionic plate, large infiltrate in chorionic plate/membranes	Inflammation present in the wall of 2 vessels (vein and artery)	2–3 sections with each 1 focus of chronic inflammation of >5 villi
3	Same as 2, including micro-abscesses in chorionic plate	Inflammation present in the wall of 3 vessels (vein and 2 arteries)	3 sections with each >2 foci of chronic inflammation of >5 villi

PMNs polymorphonuclear cells.

### Amniotic fluid analysis

After macroscopic assessment, the samples were purified by filtration (70 µL filter, Falcon BD) and centrifugation (1500 RPM, 10 minutes) to remove debris and blood clots. The supernatant was filtered through a 0.2 µL sterilization filter (Falcon, BD) to remove leukocytes and other contaminants. The sterile, acellular fraction of amniotic fluid was stored at −80°C until further analysis. Enzyme linked immunosorbent assays (ELISA) were used to determine interleukin-6 (IL-6), interleukin-8 (IL-8) and tumor necrosis factor-α (TNF-α) concentrations (CLB, Sanquin, Amsterdam, The Netherlands). The lower limits of detection for IL-6, IL-8 and TNF-α were 0.006 ng/mL, 0.01 ng/mL and 0.014 ng/mL, respectively. IL-6 and IL-8 concentrations were never undetectable. Amniotic fluid cellular composition was not systematically determined.

### Amniotic fluid pilot experiments

Several pilot experiments were conducted to validate the methods of amniotic fluid analysis.

(a) The influence of storage at +4°C during 72 hours was studied in paired fresh and stored samples. (b) To minimize the effect of aspecific binding proteins, spiking of diluted amniotic fluid with synthetic cytokines was performed to find the optimal dilution for ELISA. (c) Intra-assay and interassay reproducibility were determined by comparing the results of duplo amniotic cytokine measurements within and between ELISA assays. (d) The effect of different intervals from labor until amniotic fluid sampling or from sampling until delivery between individuals was studied by collection and analysis of serial samples. (e) The influence of delay of amniotic fluid sampling after spontaneous rupture of membranes was analysed by comparing deliveries with different delay intervals. (f) To study whether transvaginal collection of amniotic fluid affects cytokine concentrations, paired samples were collected transvaginally and using an intrauterine catheter simultaneously.

### Statistical analysis

The association between intrauterine inflammation and term vaginal delivery was studied. This cross-sectional study was part of a larger birth cohort study, for which a sample size of 500 participants was calculated; a sample size calculation for this study was not performed. Logarithmic transformation was used for amniotic fluid IL-6 and IL-8 concentrations, to provide a normal distribution. TNF-α was dichotomized into detectable and undetectable, because a large proportion of the samples contained undetectable levels of TNF-α. The frequency of chorioamnionitis, funisitis and villitis was compared between term vaginal deliveries and elective CS using the Pearson's χ^2^ or Fisher's Exact test. The geometric mean amniotic fluid IL-6 and IL-8 concentrations were compared using the Student's T test. The proportion of detectable TNF-α was compared using the Pearson's χ^2^ test. Unadjusted odds ratios (OR) of chorioamnionitis, funisitis, villitis, amniotic fluid IL-6, IL-8 and detectable TNF-α for term vaginal delivery were calculated. With multivariable logistic regression analysis, OR were adjusted for potential confounders, i.e. maternal age, gestational age, parity, meconium-staining. These potential confounders were selected for their biological plausibility and for their correlation to both intrauterine inflammation and term vaginal delivery. Collinearity between amniotic fluid cytokine concentrations was studied by calculating correlation coefficients. All data were analysed in the Statistical Package for Social Sciences (SPSS) version 15.0.

## Results

### Study population

**Figure 1:**
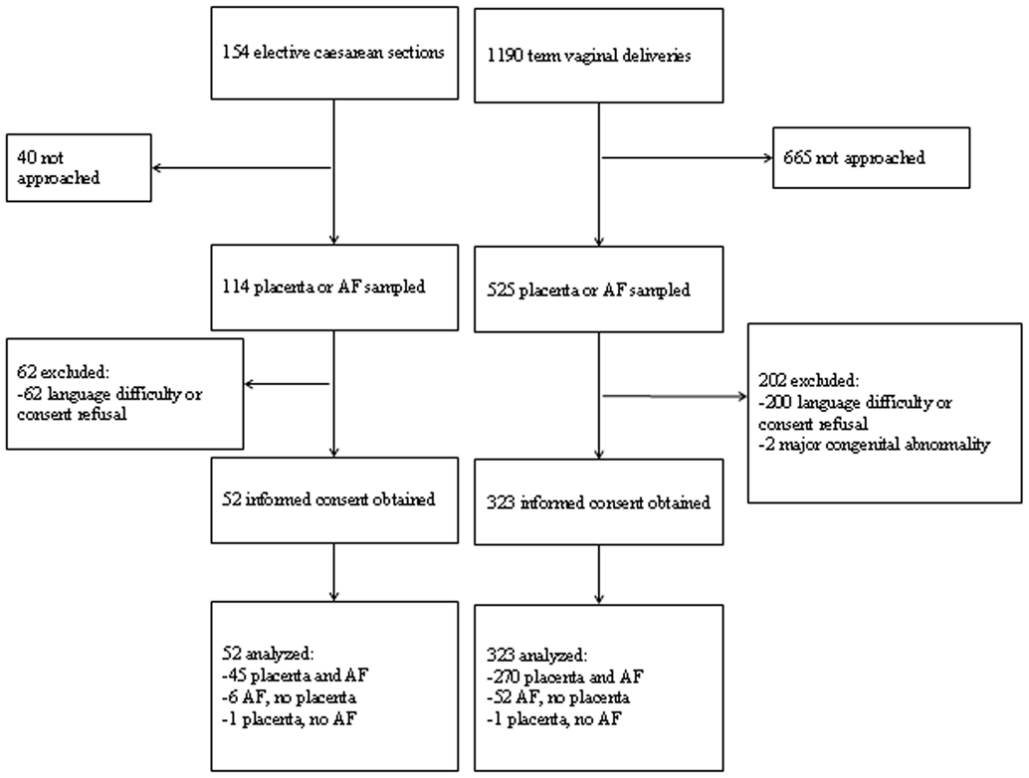
Flow chart of women participating in the study. Amniotic fluid (AF).

During the study period, 1190 term vaginal deliveries occurred.and 154 elective CS were performed. Informed consent was obtained from 375 mothers.(figure 1) Clinical characteristics of the excluded mothers and their children were similar to the characteristics of those included (data not shown).

### Clinical characteristics

The women with spontaneous onset of labor, were younger, had a higher gestational age and a higher proportion of meconium stained amniotic fluid, as compared to the women in the elective CS group (table 2). Maternal fever during labor only occurred in 4 cases (1%). Newborn infection within 48 hours after birth did occur in one child (0.3%). Within the group of women with spontaneous onset of labor, 18 of the 323 women (6%) were delivered by vacuum extraction and another 12 (4%) by secondary CS. No mother reported antenatal corticosteroid administration. The majority of elective CS was performed because of a previous CS in the past obstetric history (18/52 (35%)), breech presentation (17/52 (33%)) or placenta or pelvic pathology, such as placenta previa (10/52 (19%)).

**Table 2:** Clinical characteristics of deliveries.

Characteristic	Elective CS (n = 52)	Spontaneous onset of labor (n = 323)	P-value
Mother			
Age mother (years)^A^	34.3 (30.6 – 36.7)	31.9 (28.3 – 34.8)	0.03^B^
Gestational age at delivery (weeks)^A^	39.0 (38.5 – 39.3)	40.1 (39.3 – 40.9)	<0.001^B^
Nulliparity	15 (29%)	143 (44%)	0.04^C^
Meconium stained amniotic fluid	0 (0%)	57 (18%)	0.001^C^
Fever during delivery	0 (0%)	4 (1%)	0.999^D^
Child			
Birth weight (g)^A^	3571 (3278 – 4006)	3580 (3265 – 3835)	0.46^B^
Male gender	23 (44%)	178 (55%)	0.14^C^
Apgar score <7 after 5 minutes	3 (6%)	1 (0.3%)	0.008^D^
Newborn infection	0 (0%)	1 (0.3%)	0.999 D

CS caesarean section.

A Median value (interquartile range).

B Mann-Whitney U test.

C χ^2^-test.

D Fisher's Exact test.

### Placenta histology

**Figure 2:**
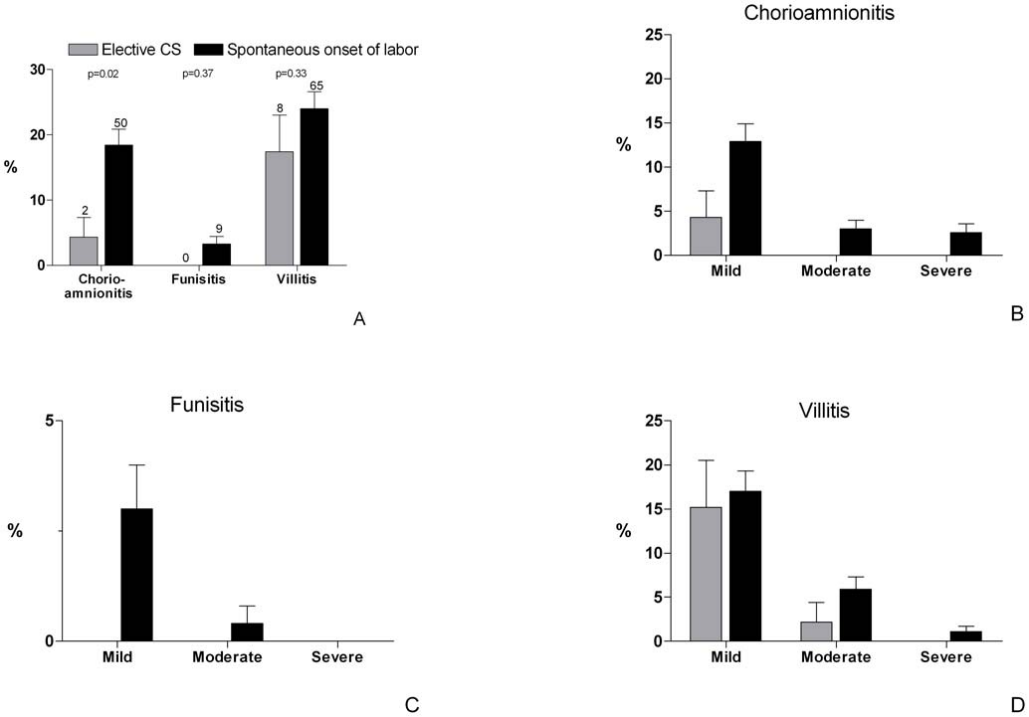
Prevalence and severity of signs of placenta inflammation. Presence of chorioamnionitis, funisitis and villitis of the placenta in elective caesarean sections (CS, n = 46) and spontaneous onset of labor (n = 271) (absolute numbers above bars).

Placenta histology was established in 317 samples. The remaining 58 placentas were not collected for non-specific reasons. Chorioamnionitis was present in 50 of 271 placentas (18%) in the group of women with spontaneous onset of labor and in 2 of 46 placentas (4%) in the elective CS group (difference 14.1%, 95% confidence interval (CI) 6.6–21.6, p = 0.02) (figure 2). Funisitis was rare in this study, but associated with chorioamnionitis in all cases (fraction funisitis in chorioamnionitis: 9/50 (18%)). Placentas with chorioamnionitis showed villitis in 27% (14/52). The prevalence of villitis was not related to gestational age at delivery. The prevalence of chorioamnionitis was higher in women who delivered for the first time as compared to multiparous women (27% vs 13%, p = 0.002).

### Amniotic fluid inflammation

**Figure 3:**
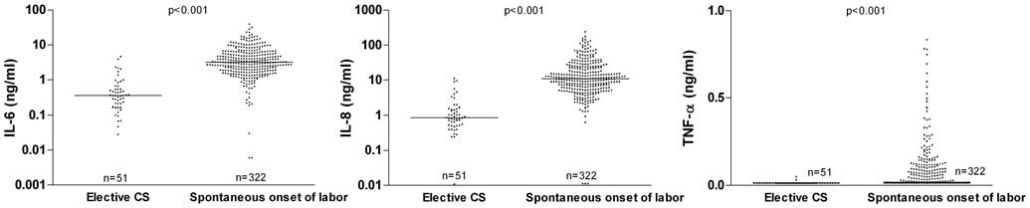
Amniotic fluid proinflammatory cytokine concentrations. Interleukin 6 (IL-6), IL-8 and tumor necrosis factor α (TNF-α) concentration in amniotic fluid samples of elective caesarean sections (CS, n = 51) and spontaneous onset of labor (n = 322) (horizontal line representing geometrical mean).

Amniotic fluid cytokines were determined in 373 samples. In the group of women with spontaneous onset of labor, amniotic fluid IL-6 concentrations were higher than in elective CS (3.1 vs 0.37 ng/mL; ratio 8.41, 95% CI 6.15–11.51, p<0.001, figure 3). Similar differences were found for amniotic fluid IL-8 concentrations (10.93 vs 0.96 ng/mL; ratio 11.34, 95% CI 7.90–16.27, p<0.001) and proportion of detectable TNF-α (50 vs 4%; difference 45.8%, 95% CI 38.1–53.4, p<0.001). Similar results were found when women with maternal fever and/or instrumental vaginal delivery or secondary CS were excluded from the analysis. Subgroup analysis did not reveal differences in cytokine concentrations for gestational age, meconium-staining or gender (data not shown). IL-6 was higher in women who delivered for the first time as compared to multiparous women (3.97 vs 2.77 ng/mL, p = 0.001). This marginal difference could not explain the contrast between the spontaneous onset of labor group and the elective CS group.

### Multivariable analysis of the association between intrauterine inflammation and term spontaneous onset of labor

Multivariable logistic regression analysis of placenta and amniotic fluid inflammation associated with term vaginal delivery was performed (table 3). Crude OR were adjusted for potential confounders, i.e. maternal age, gestational age, parity and meconium-staining. Chorioamnionitis and villitis were not independently associated with term vaginal delivery. Funisitis was not analysed, because of the low prevalence. Amniotic fluid IL-6, IL-8 and TNF-α were independently positively associated with term vaginal delivery.

**Table 3:** Multivariable analysis of placenta and amniotic fluid inflammation associated with term vaginal delivery.

	Odds ratio (95% CI)	
Factor	Unadjusted	Adjusted^A^
***Placenta (n = 317)***		
Chorioamnionitis	4.98 (1.17 – 21.22)	3.30 (0.73 – 14.81)
Funisitis	NA	NA
Villitis	1.50 (0.67 – 3.38)	1.79 (0.72 – 4.46)
***Amniotic fluid (n = 373)***		
Log IL-6	31.01 (13.37 – 71.94)	31.45 (11.83 – 83.67)
Log IL-8	33.74 (14.17 – 80.33)	26.48 (10.22 – 68.61)
TNF-α detectable	24.20 (5.79 – 101.18)	18.40 (4.32 – 78.33)

OR odds ratio, CI confidence interval, NA not applicable, IL interleukin, TNF-α tumor necrosis factor-α.

A Adjusted for potential confounders: maternal age, gestational age, parity, meconium-staining.

### Correlation between placenta and amniotic fluid inflammation

Amniotic fluid IL-6 and IL-8 concentrations were correlated (Pearson's ρ = 0.61, p<0.001), suggesting that these cytokines play a role in the same inflammatory event, but do not have an identical expression pattern. Chorioamnionitis was only weakly correlated with amniotic fluid IL-6, IL-8 and TNF-α (Spearman's ρ = 0.23, p<0.001, ρ = 0.22, p<0.001, and ρ = 0.17, p = 0.003, respectively).

### Amniotic fluid pilot experiments

**Figure 4:**
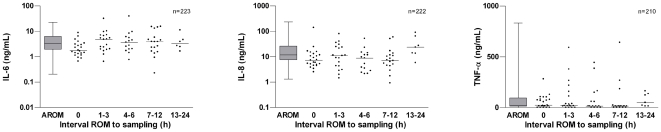
Interval between ROM and amniotic fluid sampling in relation to amniotic fluid inflammation. Relation of the interval between spontaneous rupture of membranes (ROM) and sampling and amniotic fluid interleukin 6 (IL-6), IL-8, and tumor necrosis factor α (TNF-α) concentrations in women with spontaneous onset of labor. The results of samples collected at artificial ROM (AROM) are shown in a boxplot (n = 142). ANOVA with Bonferroni correction did not show differences between the groups shown.

(a) Amniotic fluid cytokine concentrations measured in fresh samples were similar after storage. (b) Spiking of amniotic fluid with synthetic cytokines showed good reliability of dilution of amniotic fluid (n = 7, ρ = 0.93, p = 0.003). (c) Amniotic fluid cytokine measurements had a high intra-assay and interassay reproducibility (intra-assay duplicates n = 145, ρ = 0.99, p<0.001, interassay duplicates n = 6, Wilcoxon's p = 0.12). (d) We did not find an association between duration of labor until sampling, or duration from sampling until delivery and cytokine concentrations in amniotic fluid (labor until sampling n = 229, ρ = 0.06, p = 0.36, sampling until delivery n = 251, ρ = −0.01, p = 0.83). (e) Delay of sampling after spontaneous rupture of membranes did not result in different amniotic fluid cytokine concentrations (figure 4). (f) Amniotic fluid cytokine concentrations in samples simultaneously collected transvaginally and using an intrauterine catheter were similar and highly correlated (n = 13, ρ = 0.81, p<0.001).

## Discussion

In this cross-sectional study of 375 term vaginal deliveries and elective CS, we found that signs of intrauterine inflammation are associated with spontaneous onset of labor. Histological signs of chorioamnionitis were found in 18% of placentas of term vaginal deliveries, whereas acute inflammation was virtually absent in elective CS. Amniotic fluid IL-6 and IL-8 concentrations and the proportion of deliveries with detectable amniotic fluid TNF-α were approximately tenfold higher in term vaginal deliveries than in elective CS.

Intrauterine inflammation has been shown to induce preterm labor in a primate model. Intra-amniotic injection of TNF-α and IL-1β rapidly induced onset of labor.[9] The current study suggests that intrauterine inflammation might play a role in the physiological process of onset of labor at term gestation in humans. Presumably, the contractility, excitability and connectivity of uterine myometrical cells is upregulated upon triggering by amniotic proinflammatory cytokines.[17] In addition, cervical ripening and dilatation can be promoted by exposure to increased levels of inflammatory cytokines.[18]–[20] For example, IL-8 increases activity of collagenase and metalloproteinases 8 and 9, which are required for softening of cervical tissue. Towards the end of the pregnancy in humans, maternal expression of circulating corticotropin-releasing hormone (CRH) increases exponentially, which is accompanied by increased excitability the myometrium allowing the initiation of contractions.[21] It is conceivable that intrauterine inflammation has a direct positive effect on these processes that characterize the pre-partum phase.

The origin of intrauterine inflammation at term is uncertain but might be infectious. An estimate of 18% of women will have microbial invasion of the amniotic cavity at term, increasing to 30% when there is prolonged rupture of the membranes.[6], [7], [13], [14] Indolent pathogens, like *Ureaplasma Urealyticum*, have been associated with the onset of labor.[22]–[24] Many of these pathogens are not isolated by conventional bacterial cultures.[25], [26] Placenta histological examination showed chorioamnionitis and occasionally funisitis, predominantly in the spontaneous onset of labor group. These cases might represent the maternal and fetal response to a microbial invasion of the amniotic cavity. During preterm labor, chorioamnionitis has been associated with increased amniotic fluid inflammatory cytokine concentrations, but not during preterm prelabor rupture of membranes.[27] By contrast, a relation between villitis and the onset of labor is not likely, because of the non-acute character of villitis. [28]–[30] Moreover, the group of women with spontaneous onset of labor and the elective CS group showed similar villitis prevalences. Inflammation at term could also be of fetal origin. Surfactant protein A (SP-A) is produced by macrophages in the fetal airways. SP-A in the amniotic fluid promotes an inflammatory response in the chorioamniotic membranes. In the mouse model, intrauterine SP-A administration results in prompt labor.[31] More research is required to establish the relative maternal and fetal contribution to inflammation at term.

The major strengths of this study are that it examined evidence of inflammation in different intrauterine compartments and the large sample size. Amniotic fluid samples were collected vaginally during active labor. Other studies have collected amniotic fluid by amniocentesis or in pregnant women presenting with preterm contractions.[2], [3], [7], [14], [23], [25], [27], [32], [33] Furthermore, our sample of women with spontaneous onset of labor essentially consisted of uncomplicated pregnancies (e.g. no use of antenatal steroids) and deliveries (e.g. 90% unassisted physiologic deliveries).

Some possible limitations should also be discussed. The cross-sectional design of data collection does not allow definite conclusions on causality. The inflammation might also be the result of birth process, including maternal stress by labor activity or fetal stress by exposure to contractions during labor and passage through the birth canal.[34] However, the presence of histological signs of inflammation in the placenta suggests that inflammation precedes delivery. The modes of amniotic fluid sampling that were distinct for the two groups studied might have contributed to the differences found. But women with secondary CS after the onset of labor and women who delivered vaginally had similar high levels of amniotic fluid inflammation (e.g. IL-6 3.49 ng/mL vs 3.25 ng/mL, p = 0.82). We excluded the potential bias that vaginal collection of amniotic fluid, as compared to the gold standard of intraamniotic collection, might have introduced. A delay in sampling of amniotic fluid after spontaneous rupture of membranes did not influence cytokine concentrations. A number of possible neonatal correlations (e.g. tracheal aspirates, cord blood acidosis) were not studied, for this study sample consisted of healthy newborns.

The results of this study may have clinical implications. Better insight in the physiological role of intrauterine inflammation may eventually result in strategies to intervene in normal labor and delivery, including induction of labor in postterm pregnancies.[20], [35] We have considered the potential physiological role of intrauterine inflammation for both the onset of spontaneous labor at term and the development and maturation of fetal and neonatal airways.[9], [32], [34], [36]–[39] The candidate role of intrauterine inflammation as a downstream step of the corticosteroid pathway, which is known to be involved in airway maturation, is intriguing.

### Conclusions

In conclusion, human term spontaneous labor and delivery are associated with a high level of intrauterine inflammation, and intrauterine inflammation is a consistent finding in all intrauterine compartments. This large cross-sectional study shows that normal labor at term is characterized by chorioamnionitis in a considerable number of cases and high amniotic fluid concentration of proinflammatory cytokines in all cases, providing insight in the pivotal role of intrauterine inflammation in normal parturition.
